# Progress of the National Insurance Bill

**Published:** 1911-06-17

**Authors:** 


### PROGRESS OF THE NATIONAL INSURANCE BILL.

The House of Commons reassembled on June 13, and will adjourn on the evening of June 20 till Monday, June 26. This week the House is mainly occupied with financial business, and the committee stage of the Insurance Bill will not be reached until after the Coronation. Several questions having reference to the Bill were asked on June 13, one of which related to the position of hospitals. The question was put by Lord Balcarres on behalf of Mr. Barlow, who desired to know whether, in view of the fact that, under the provisions of the Bill, various hospitals and charitable institutions now supported by voluntary public contributions would be called upon to find additional expenditure necessitated by the insurance of nurses, attendants, etc., (whilst at the same time additional work was being placed upon them by the certain increase of patients consequent on universal medical attendance and the probable withdrawal of public voluntary support consequent on the introduction of such an Act as the National Insurance Bill), the Chancellor could see his way to assist, from the moneys received under such a Bill, towards the upkeep of such hospitals and charitable institutions. Mr. Lloyd George, in his reply, said the hon. member had apparently overlooked clause 17 of the Bill, which enables approved societies to subscribe to hospitals and other charitable institutions; he thought the position of such institutions was likely, on the whole, to be strengthened rather than prejudiced by the operation of the Bill.

In his speech at Birmingham on Saturday last, the Chancellor of the Exchequer said he hoped to see the scheme an Act of Parliament within the next three months. If this is accomplished, discussion of the details of the scheme will necessarily be curtailed, for the House has before it a considerable amount of other important business which will occupy a good deal of time. If the Bill is not passed by the end of August there will probably be an autumn session, but failing that, the Bill will have to be carried over to next year. In Saturday's speech the Chancellor referred again to the position of medical practitioners, but he said nothing that will help to settle existing difficulties. He said: "The doctors say to me, 'Six shillings is not enough.' . . Friendly Societies say to me, 'How dare you give as much?' . . The only comment I would make is this: When one set of people say you are paying too little and another set of people think you are paying too much, it rather means you are somewhere about right. However, may I say this to my friends of the Friendly Societies: A badly-paid service is a bad service, and there is no business where an adequate, a fair remuneration is more essential than in the profession of healing. A man ought to enter your sick room with a sense, at any rate, that he is fairly treated. I am confident that that difficulty can be overcome." The amendments to be moved on behalf of the British Medical Association have not yet been put on the paper, but a number of proposed amendments are being drafted.

